# Time to catch-up: postnatal growth trajectories in preterm infants at a tertiary care hospital in Riyadh, Saudi Arabia

**DOI:** 10.1186/s12887-026-07223-5

**Published:** 2026-06-24

**Authors:** Mahdi A. Alnamnakani, Lana A. Shaiba, Adnan Hadid, Reem Alshathri, Abdulrahman Alaujan, Amal Alshibi, Haifa Alwael, Maha Babtain, Abdulrahman Al Wallan, Jawahir M. Abuhaimed

**Affiliations:** 1 Department of Pediatrics, College of Medicine, King Saud University, P.O. Box 12372, Riyadh, Saudi Arabia; 2 Department of Neonatology, King Saud University Medical City, Riyadh, Saudi Arabia; 3 Department of Pediatrics, King Saud University Medical City, Riyadh, Saudi Arabia; 4 Department of General Pediatric Medicine, King Fahad Medical City, Riyadh, Saudi Arabia; 5 Department of Pediatrics, King Abdullah Specialized Children’s Hospital, Ministry of National Guard and Health Affairs, Riyadh, Saudi Arabia; 6 College of Medicine, Alfaisal University, Riyadh, Saudi Arabia; 7 Pediatric Department, Children’s Specialized Hospital, King Fahad Medical City, Riyadh, Saudi Arabia

**Keywords:** Preterm infant, Postnatal growth, Catch-up growth, Birth weight, Saudi Arabia

## Abstract

**Background:**

Preterm birth remains a significant global health concern, with long-term outcomes closely linked to postnatal growth trajectories. Data regarding the timing of catch-up growth in preterm infants from Middle Eastern populations are limited. This study aimed to assess the time required to achieve catch-up weight and to characterize postnatal growth trajectories across birth-weight categories at a tertiary care hospital in Riyadh, Saudi Arabia.

**Methods:**

This retrospective cohort study included 134 preterm infants (< 37 weeks’ gestational age) and 193 term infants born between January 2018 and early 2019 at King Saud University Medical City. Anthropometric measurements were recorded at predefined intervals up to 24 months of corrected age. Growth was evaluated using sex-specific Z-scores based on WHO Child Growth Standards. Catch-up growth was defined as a Z-score increase > 0.67 between two consecutive time points. Longitudinal weight and length trajectories were analyzed using linear mixed-effects models with subject-specific random intercepts and a first-order autoregressive covariance structure, with adjusted estimated marginal means derived over time. Reporting followed the STROBE statement for cohort studies.

**Results:**

Preterm infants had significantly lower birth weight (1.72 ± 0.60 kg vs. 2.97 ± 0.66 kg; *p* < 0.001) and birth length (40.65 ± 5.36 cm vs. 48.27 ± 3.55 cm; *p* < 0.001) compared with term infants. In mixed-effects models, preterm infants had lower adjusted weight overall (*p* = 0.006), but the group-by-age interaction was not significant (*p* = 0.271), indicating parallel weight gain over time. For length, a significant group-by-age interaction (*p* < 0.001) indicated that linear-growth trajectories differed between groups, with preterm infants remaining shorter throughout follow-up. Weight-for-age Z-scores converged across birth-weight categories by 24 months. Catch-up growth rates were highest among very low birth weight infants (31.8% by 24 months).

**Conclusions:**

Preterm infants demonstrate significant early growth deficits with weight catch-up achieved within the first year of corrected age, while linear growth recovery extends closer to 24 months. These findings underscore the importance of vigilant growth monitoring and individualized nutritional strategies during early infancy.

**Supplementary Information:**

The online version contains supplementary material available at https://doi.org/10.1186/s12887-026-07223-5.

## Background

Preterm birth remains a major global public health concern, with an estimated 14.9 million infants born prematurely each year worldwide, accounting for approximately 11% of all live births [1]. Despite substantial advances in neonatal intensive care and improved survival rates, complications related to prematurity continue to contribute to nearly one-third of neonatal deaths globally [2]. Beyond mortality, preterm infants are at increased risk of both in-hospital complications and long-term adverse outcomes, many of which are closely linked to postnatal growth trajectories and early nutritional status.

Adequate postnatal growth and timely achievement of catch-up weight are widely regarded as key indicators of recovery and overall health in preterm neonates. Numerous studies have demonstrated that preterm infants have significantly lower birth weight and length compared with term infants, differences that often persist after hospital discharge and during subsequent follow-up visits, particularly among extremely preterm infants and those born small for gestational age [3–5]. Failure to achieve appropriate catch-up growth has been associated with higher rates of rehospitalization, impaired neurodevelopment, and unfavorable metabolic outcomes later in life [6–8]. Moreover, postnatal growth failure remains a common challenge among preterm infants, especially those born at earlier gestational ages or with low birth weight, and may persist well beyond infancy [9].

While a growing body of literature has examined growth outcomes in preterm infants, data regarding the timing required to achieve catch-up growth remain limited, particularly in Middle Eastern populations. Growth trajectories may be influenced by regional variations in neonatal care practices, nutritional strategies, and post-discharge follow-up protocols. In Saudi Arabia, despite a high standard of neonatal care, local evidence describing post-discharge growth patterns and catch-up timing among preterm infants is scarce.

Therefore, this study aims to assess the time required to achieve catch-up weight among infants born prematurely at a tertiary care hospital in Riyadh, Saudi Arabia, and to characterize postnatal growth trajectories across different birth-weight categories.

## Methods

### Study design and setting

This was a retrospective cohort study conducted at King Saud University Medical City (KSUMC), a tertiary hospital in Riyadh, Saudi Arabia. Infants born between January 2018 and early 2019 who received routine follow-up care at the institution were eligible for inclusion. The study was not prospectively registered, as it is a retrospective analysis of anonymized electronic medical records and no intervention was applied. Clinical trial number: not applicable.

Follow-up visits in this retrospective cohort were drawn from routine clinic attendance at predefined age windows rather than from a rigid research-driven schedule. As a result, the number of infants contributing data varies across intervals, and the resulting missingness is reported and discussed below.

### Study population and eligibility criteria

The exposed cohort consisted of preterm infants born at < 37 weeks’ gestational age (GA). A comparison group of infants born at ≥ 37 weeks’ GA during the same study period was included. Gestational age was determined from the mother’s last menstrual period or first-trimester ultrasonogram.

Infants were followed from birth until 24 months of age. Exclusion criteria included major congenital malformations, chromosomal abnormalities, or known genetic syndromes, as well as inadequate follow-up, defined as attendance at fewer than three routine follow-up visits during the study period.

### Data collection

Anthropometric data including weight and length were obtained retrospectively from the electronic medical records of KSUMC. Measurements were recorded at predefined age intervals: 2–4, 4–6, 6–8, 8–12, and 24 months. Head circumference was recorded at birth but was not consistently documented at subsequent visits in the electronic records; for this reason, longitudinal analysis of head circumference was not feasible and is acknowledged as a limitation.

During admission, preterm infants were predominantly fed expressed breast milk supplemented with human milk fortifier (commonly at a 1:25 ratio) and/or preterm formula, provided on an ad libitum or scheduled (approximately every 2–3 h) basis according to tolerance. After discharge, infants were transitioned to standard or post-discharge formula and breast milk, with complementary foods introduced at the 4–6 months of life. Detailed quantitative macronutrient intake was not available from the records, which is acknowledged as a limitation.

### Definitions

Preterm birth was defined as birth before 37 completed weeks of gestation, while term birth was defined as birth at ≥ 37 weeks’ GA. Birth weight categories were defined as follows: Very low birth weight (VLBW): <1500 g; Low birth weight (LBW): <2500 g; Normal birth weight (NBW): ≥2500 g.

Corrected age (CA) was calculated for every preterm infant by subtracting the number of weeks born before 40 weeks’ gestation from the chronological age, and all Z-score calculations and growth-trajectory comparisons for preterm infants were performed using corrected age throughout the full 24-month follow-up period.

Catch-up growth was defined as an increase in weight-for-age Z-score greater than 0.67 between two consecutive time points for the same infant (a within-infant, paired change), consistent with previously published criteria [10]. An increment of approximately 0.67 SD corresponds to crossing one percentile band on standard growth charts and is the conventional threshold used to define clinically meaningful catch-up. Poor growth was defined as a weight-for-length Z-score (WLZ) below − 2 standard deviations.

### Outcome measures

The primary outcome was the achievement of catch-up growth in preterm infants compared to term infants. Growth was evaluated using weight and length measurements converted to sex-specific Z-scores based on corrected age, using the World Health Organization (WHO) Child Growth Standards. Z-scores were computed using the WHO Anthro software (version 3.2.2; World Health Organization, Geneva, Switzerland).

A single growth reference (the WHO Child Growth Standards) was applied on corrected age across the entire 24-month follow-up to allow internally consistent Z-score comparisons over time and directly between preterm and term infants on the same scale. We recognize that the Fenton and INTERGROWTH-21st standards are better suited to the early neonatal and in-hospital period; this consideration is noted in the Limitations.

### Sample size

Due to the retrospective nature of the study, a convenience sample of eligible infants with complete longitudinal data was used. A total of 134 preterm infants met the inclusion criteria and were included in the analysis, exceeding the minimum estimated sample size requirement for detecting between-group Z-score differences of 0.4 SD at 80% power and α = 0.05.

### Statistical analysis

Data were analyzed using the Statistical Package for the Social Sciences (SPSS version 26.0; IBM Corp., Armonk, NY, USA). Continuous variables are presented as mean ± standard deviation (SD), and categorical variables as frequencies and percentages. Baseline characteristics of preterm and term infants were compared using independent-samples t-tests for continuous variables and chi-square tests for categorical variables.

To evaluate longitudinal changes in anthropometric outcomes and account for the repeated measurements obtained from the same infant over time, linear mixed-effects models were fitted for weight and length trajectories. Fixed effects included group (preterm vs. term), corrected age, sex, and the interaction between group and corrected age. Subject-specific random intercepts were included to account for within-infant correlation, and a first-order autoregressive [AR(1)] covariance structure was specified for the repeated measurements. Parameters were estimated using restricted maximum likelihood (REML), and denominator degrees of freedom were calculated using the Satterthwaite approximation.

Additional mixed-effects models evaluated weight-for-age and length-for-age Z-score trajectories according to birth-weight category (very low birth weight [VLBW], low birth weight [LBW], and normal birth weight [NBW]). These models included birth-weight category, corrected age, sex, gestational age, and the interaction between birth-weight category and corrected age as fixed effects, with subject-specific random intercepts and an AR(1) covariance structure. Adjusted estimated marginal means with 95% confidence intervals (CIs) were derived from the models to describe growth trajectories over time, and pairwise comparisons were adjusted using the Bonferroni method where applicable.

Catch-up growth was defined as an increase in weight-for-age Z-score greater than 0.67 between two consecutive time points for the same infant. Rates of catch-up growth were compared between birth-weight groups using chi-square or Fisher’s exact tests, as appropriate.

Linear mixed-effects models were estimated using all available longitudinal observations under the assumption that data were missing at random, reducing potential bias from incomplete follow-up and avoiding the need for imputation. The number of infants contributing data at each follow-up interval is reported in the corresponding tables. All statistical tests were two-sided, and a p-value < 0.05 was considered statistically significant. Reporting of this study adheres to the STROBE statement for cohort studies [11], and a completed STROBE checklist is provided as Additional file 1.

Prior to analysis, the dataset was screened for biologically implausible values. A small number of weight records containing misplaced-decimal data-entry errors (single values such as 68.0, 42.0, 36.3 and 73.1 kg in the term group and 30 and 35 kg in the preterm group) were identified by cross-checking against the source records and corrected. After correction, all weight distributions were biologically plausible, resolving the previously inflated standard deviations.

### Ethical considerations

Ethical approval was obtained from the Institutional Review Board of King Saud University Medical City (IRB No. E-21-6275, approved on 10 October 2021). Given the retrospective nature of the study and the use of de-identified medical records, the requirement for informed consent was waived by the ethics committee.

## Results

### Baseline characteristics

A total of 327 infants were included, comprising 134 preterm and 193 term infants. Baseline characteristics are summarized in Table 1. Preterm infants had significantly lower gestational age (31.57 ± 3.14 vs. 38.15 ± 1.59 weeks, *p* < 0.001), birth weight (1.72 ± 0.60 vs. 2.97 ± 0.66 kg, *p* < 0.001), birth length (40.65 ± 5.36 vs. 48.27 ± 3.55 cm, *p* < 0.001), and head circumference (29.27 ± 2.98 vs. 33.97 ± 2.08 cm, *p* < 0.001) than term infants. The proportion of male infants was similar between groups (52.2% vs. 57.0%, *p* = 0.388). Antenatal records documented fetal growth restriction (small-for-gestational-age or intrauterine growth restriction) in approximately 9% of the preterm cohort; given the small absolute number, gestational age was carried as a covariate in the Z-score models rather than analyzing these infants as a separate subgroup.

**Table 1:** Baseline characteristics of preterm and term infants

Variable	Preterm (*n* = 134)	Term (*n* = 193)	*p*-value
Gestational age (weeks)	31.57 ± 3.14	38.15 ± 1.59	< 0.001
Birth weight (kg)	1.72 ± 0.60	2.97 ± 0.66	< 0.001
Birth length (cm)	40.65 ± 5.36	48.27 ± 3.55	< 0.001
Head circumference (cm)	29.27 ± 2.98	33.97 ± 2.08	< 0.001
Male sex, n (%)	70 (52.2)	110 (57.0)	0.388

*SD* Standard deviation

*p*-values for continuous variables by independent t-test; categorical by chi-square

Results of the linear mixed-effects model for longitudinal weight are presented in Table 2. Corrected age was strongly associated with weight gain over time (F = 296.436, *p* < 0.001). Preterm infants had significantly lower adjusted body weight than term infants overall (F = 7.739, *p* = 0.006). The interaction between corrected age and group was not statistically significant (F = 1.278, *p* = 0.271), indicating similar patterns of weight gain over time in both groups after accounting for repeated measurements. Male infants had significantly higher weight than females throughout follow-up (F = 15.417, *p* < 0.001).

**Table 2:** Linear mixed-effects model for longitudinal weight

Effect	F	df (numerator, denominator)	*p*-value
Time	296.436	(5, 1045.077)	< 0.001
Group (preterm vs. term)	7.739	(1, 345.478)	0.006
Time × Group	1.278	(5, 1045.544)	0.271
Sex	15.417	(1, 328.858)	< 0.001

Linear mixed-effects model with AR(1) covariance structure and random intercept per subject; outcome: weight (kg) across 6 time points; estimation by REML

Model-based estimated marginal means for weight are shown in Table 3. Adjusted mean weight increased progressively from 2.31 kg (95% CI 1.96–2.66) at birth to 13.56 kg (95% CI 13.00–14.12) at 24 months corrected age.

**Table 3:** Adjusted marginal means of weight over time (model-based estimates)

Age (corrected)	Adjusted mean (kg)	SE	95% CI
Birth	2.31	0.18	1.96–2.66
2–4 months	5.07	0.20	4.68–5.46
4–6 months	6.98	0.22	6.55–7.41
6–8 months	7.58	0.23	7.13–8.04
8–12 months	9.28	0.21	8.86–9.70
24 months	13.56	0.29	13.00–14.12

The linear mixed-effects model for longitudinal length is presented in Table 4. Corrected age had a significant effect on length growth (F = 1950.992, *p* < 0.001). Overall, preterm infants remained shorter than term infants throughout follow-up (F = 22.875, *p* < 0.001). A significant corrected age-by-group interaction was observed (F = 19.427, *p* < 0.001), indicating that length trajectories differed between preterm and term infants over time. Male infants demonstrated greater length than females (F = 10.388, *p* = 0.001).

**Table 4:** Linear mixed-effects model for longitudinal length

Effect	F	df (numerator, denominator)	*p*-value
Time	1950.992	(5, 595.414)	< 0.001
Group (preterm vs. term)	22.875	(1, 337.790)	< 0.001
Time × Group	19.427	(5, 595.435)	< 0.001
Sex	10.388	(1, 323.879)	0.001

Linear mixed-effects model with AR(1) covariance structure and random intercept per subject; outcome: length (cm) across 6 time points; estimation by REML

Adjusted marginal means for length are shown in Table 5. Mean length increased from 44.32 cm (95% CI 43.66–44.97) at birth to 94.41 cm (95% CI 93.47–95.36) at 24 months corrected age.

**Table 5:** Adjusted marginal means of length over time (model-based estimates)

Age (corrected)	Adjusted mean (cm)	SE	95% CI
Birth	44.32	0.34	43.66–44.97
2–4 months	56.95	0.35	56.25–57.64
4–6 months	64.06	0.38	63.31–64.81
6–8 months	68.16	0.40	67.37–68.94
8–12 months	72.51	0.38	71.76–73.25
24 months	94.41	0.48	93.47–95.36

Results of the mixed-effects model for weight-for-age Z-score trajectories are summarized in Table 6. Significant effects were observed for birth-weight category (F = 6.714, *p* = 0.001) and corrected age (F = 75.83, *p* < 0.001), and a significant birth-weight category-by-age interaction was identified (F = 3.307, *p* = 0.001), indicating that weight-for-age Z-score trajectories differed by birth-weight category over time. Neither sex nor gestational age significantly influenced these trajectories.

**Table 6:** Linear mixed-effects model of weight-for-age Z-score trajectories by birth-weight category

Effect	Numerator df	Denominator df	F	*p*
Birth-weight category	2	243	6.714	0.001
Time	4	261.8	75.83	< 0.001
Category × Time	8	236.5	3.307	0.001
Sex	1	237.5	0.534	0.466
Gestational age	1	238	0.009	0.924

Adjusted estimated marginal means are presented in Table 7. VLBW infants had the lowest weight-for-age Z-scores at birth but demonstrated progressive improvement, converging toward LBW and NBW infants by 24 months corrected age.

**Table 7:** Adjusted estimated marginal means of weight-for-age Z-score by birth-weight category

Age (corrected)	VLBW (< 1500 g)	LBW (1500–2499 g)	NBW (≥ 2500 g)
Birth	-0.482 (-0.563, -0.401)	-0.379 (-0.455, -0.304)	-0.190 (-0.325, -0.056)
4–6 months	0.072 (-0.016, 0.161)	0.006 (-0.076, 0.087)	0.068 (-0.048, 0.183)
6–8 months	0.002 (-0.082, 0.085)	0.006 (-0.071, 0.083)	0.043 (-0.075, 0.161)
8–12 months	0.125 (0.038, 0.213)	0.075 (-0.006, 0.156)	0.127 (0.001, 0.253)
24 months	0.102 (0.007, 0.196)	0.127 (0.044, 0.210)	0.255 (0.106, 0.404)

Values are estimated marginal means (95% CI) adjusted for sex and gestational age

The mixed-effects model for length-for-age Z-score trajectories is shown in Table 8. Corrected age was significantly associated with changes in length-for-age Z-scores (F = 89.295, *p* < 0.001). Although the overall effect of birth-weight category was not statistically significant (F = 1.05, *p* = 0.351), a significant birth-weight category-by-age interaction was observed (F = 7.241, *p* < 0.001), indicating differential linear growth trajectories among birth-weight groups. Gestational age was independently associated with length-for-age Z-score trajectories (F = 3.946, *p* = 0.048), whereas sex was not.

**Table 8:** Linear mixed-effects model for length-for-age Z-score trajectories

Effect	F-value	*p*-value
Birth-weight category	1.05	0.351
Time	89.295	< 0.001
Category × Time	7.241	< 0.001
Sex	0.057	0.812
Gestational age	3.946	0.048

Linear mixed-effects model with random intercept and AR(1) covariance structure; covariates: sex and gestational age

Adjusted estimated marginal means for length-for-age Z-scores are presented in Table 9. All birth-weight groups showed gradual improvement over time, with the largest relative gains among VLBW infants.

**Table 9:** Adjusted estimated marginal means for length-for-age Z-score by birth-weight category

Age (corrected)	VLBW	LBW	NBW
Birth	-0.228 (-0.250, -0.206)	-0.150 (-0.167, -0.133)	-0.088 (-0.134, -0.042)
4–6 months	0.012 (-0.012, 0.035)	-0.008 (-0.026, 0.011)	0.042 (-0.001, 0.086)
6–8 months	0.011 (-0.013, 0.036)	0.001 (-0.017, 0.020)	0.004 (-0.039, 0.048)
8–12 months	0.042 (0.020, 0.065)	0.031 (0.012, 0.049)	0.015 (-0.032, 0.062)
24 months	0.086 (0.054, 0.118)	0.067 (0.043, 0.091)	0.047 (-0.018, 0.111)

Values are estimated marginal means (95% CI) adjusted for sex and gestational age

### Rate of catch-up growth

**Fig. 1:**
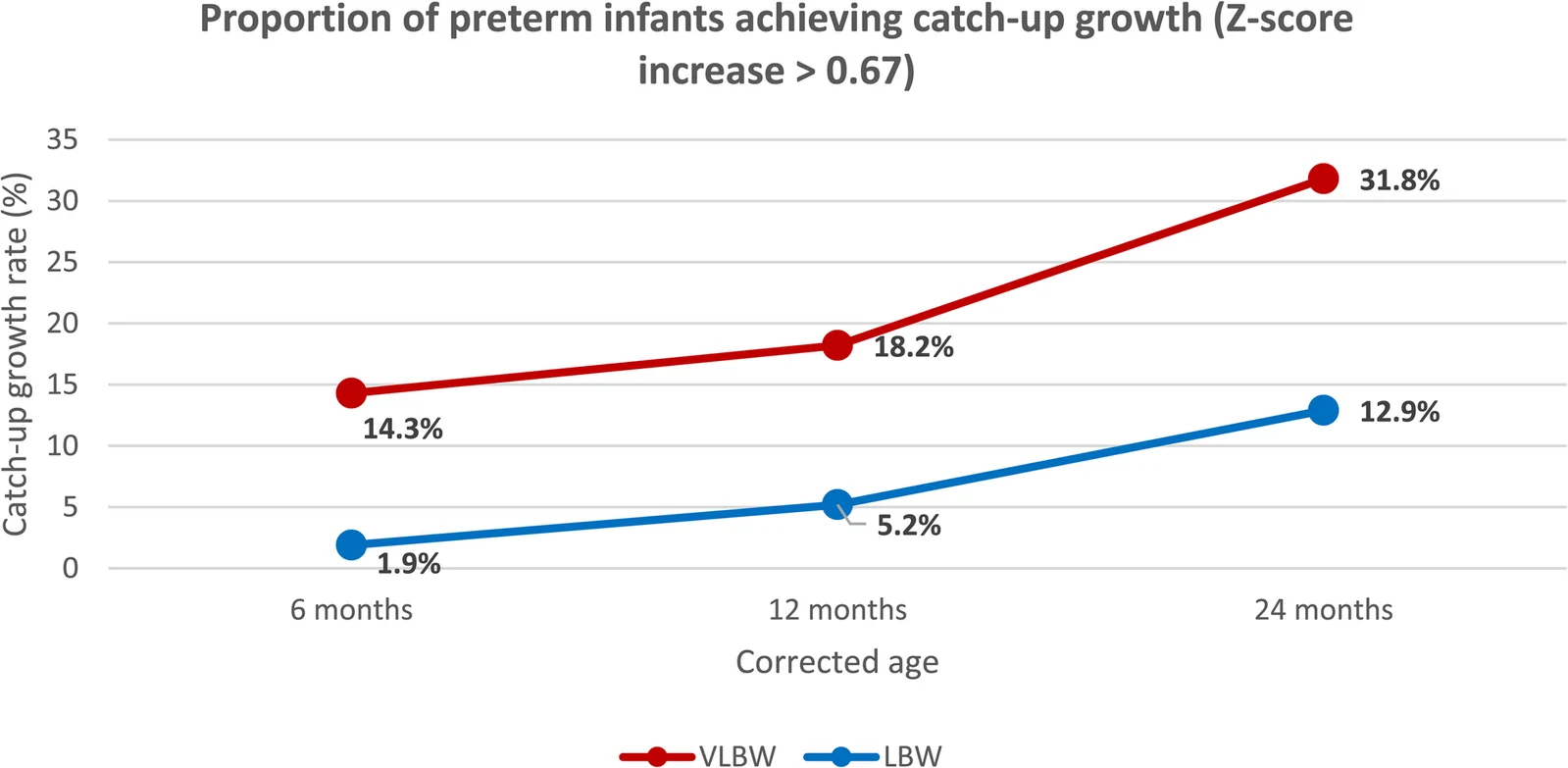
Proportion of preterm infants achieving catch-up growth (Z-score increase > 0.67) by birth-weight category across corrected age. VLBW: 14.3% (6/42) at 6 months, 18.2% (8/44) at 12 months, 31.8% (7/22) at 24 months. LBW: 1.9% (1/52), 5.2% (3/58), 12.9% (4/31). Between-group comparisons by Fisher’s exact test: *p* = 0.042, 0.052, 0.168

Rates of catch-up growth are presented in Table 10 and illustrated in Fig. 1. The proportion of infants achieving catch-up growth increased progressively with age in both birth-weight groups. Among VLBW infants, catch-up growth was observed in 14.3% at 6 months, 18.2% at 12 months, and 31.8% at 24 months corrected age; corresponding rates among LBW infants were 1.9%, 5.2%, and 12.9%. The difference between VLBW and LBW infants was statistically significant at 6 months (*p* = 0.042) but not at 12 months (*p* = 0.052) or 24 months (*p* = 0.168). Because of the small number of NBW infants (*n* = 9), comparative analyses involving this group were not performed, and the catch-up proportions in the small subgroups should be interpreted cautiously.

**Table 10:** Rate of catch-up growth (Z-score increase > 0.67 between two consecutive time points) by birth-weight category and age

Age (corrected)	VLBW, *n* (%)	LBW, *n* (%)	*p*-value
6 months	6/42 (14.3%)	1/52 (1.9%)	0.042*
12 months	8/44 (18.2%)	3/58 (5.2%)	0.052
24 months	7/22 (31.8%)	4/31 (12.9%)	0.168

**Fig. 2:**
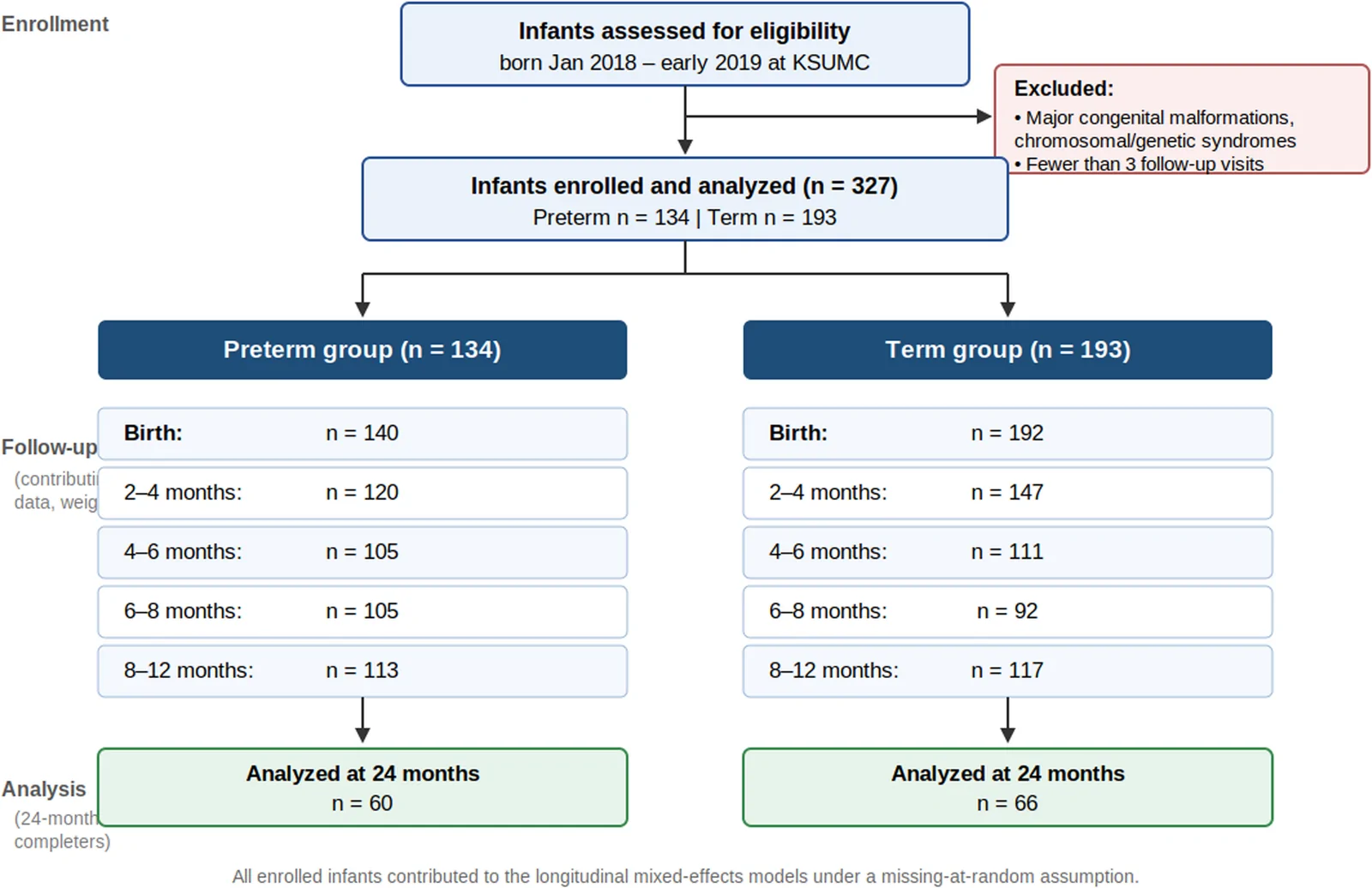
STROBE-compliant participant flow diagram showing infants assessed, excluded with reasons, enrolled (134 preterm, 193 term), the number contributing weight data at each follow-up interval, and those analyzed at 24 months of corrected age

A STROBE-compliant participant flow diagram (Fig. 2) summarizes the number of infants screened, excluded with reasons, and contributing data at each follow-up window for the preterm and term groups. As reflected in Tables 2 and 3, the number of infants contributing data declined at later intervals, particularly at 24 months. To assess whether attrition was differential, baseline birth weight and gestational age were compared between infants retained at and lost before the 24-month visit; no clinically meaningful baseline difference was identified between retained and non-retained infants. Nonetheless, the reduced sample at 24 months warrants cautious interpretation of the findings at that time point, and the potential for attrition bias is acknowledged in the Limitations.

## Discussion

This study examined postnatal growth trajectories in preterm infants, with particular attention to the timing and pattern of catch-up growth when compared with term peers and across birth-weight categories. Preterm birth remains a major global health concern and is strongly associated with early growth restriction and long-term morbidity [1, 2]. In line with this, clear differences in anthropometric measurements were evident at birth in our cohort, particularly among infants born with very low birth weight. These early disparities largely reflect the combined effects of shortened gestation and intrauterine growth restriction, which are well-established consequences of prematurity [4, 12].

Over the course of follow-up, weight trajectories in preterm infants showed progressive convergence with those of term infants. By 24 months of corrected age, mean weight values were comparable between groups. In the mixed-effects model, although preterm infants had lower adjusted weight overall, the group-by-age interaction was not significant, indicating that weight was gained at a similar rate in both groups and that the early deficit narrowed in relative terms over time. When weight was assessed using weight-for-age Z-scores, significant differences were observed during early infancy, particularly at birth and within the first 6–8 months of corrected age. These differences diminished over time, and by 24 months the adjusted marginal means converged across birth-weight groups. This pattern suggests that weight catch-up in preterm infants occurs relatively early and is largely achieved within the first year of life. Similar trends have been reported in longitudinal cohorts of extremely and very low birth weight infants, where rapid postnatal weight gain was observed during early infancy [9, 13]. Comparable findings were also described by Roberts and colleagues, who demonstrated substantial early catch-up in weight despite persistent differences later in childhood [4].

In contrast, linear growth followed a more protracted recovery course. The significant group-by-age interaction for length in the mixed-effects model confirmed that linear-growth trajectories differed between preterm and term infants over time. Length-for-age Z-scores remained significantly lower among very low birth weight infants throughout the first year of corrected age when compared with infants of higher birth weights. Unlike weight, convergence in linear growth was not observed until closer to 24 months of corrected age. This delayed recovery of length is a well-documented phenomenon in preterm populations and reinforces the concept that gains in stature tend to lag behind weight gain [3, 5]. Longitudinal studies have consistently shown that linear growth recovery extends into the second year of life and may remain incomplete in some children [4]. The persistence of these differences highlights the vulnerability of linear growth to early-life insults and nutritional limitations during critical developmental windows.

Established nutritional guidelines emphasize the importance of adequate energy, protein, and lipid intake for optimal growth in preterm infants [14, 15]. Moreover, several studies have demonstrated associations between early nutritional intake particularly protein and lipid provision and improved growth and neurodevelopmental outcomes [6–8]. In our cohort, an observed period of more rapid weight gain was noted between approximately 4 and 8 months of corrected age; we present this as a descriptive observation rather than a defined intervention window, as the observational design does not permit causal inference. Prospective studies capturing detailed macronutrient intake are needed to clarify these associations in our setting.

Overall, the findings of this study align with existing literature demonstrating that growth patterns in preterm infants differ substantially from those of term infants and are strongly influenced by gestational age and birth weight [3, 5]. Persistent alterations in growth trajectories during infancy have been linked to later neurodevelopmental and functional outcomes, particularly among those born very preterm or with extremely low birth weight [16, 17]. Collectively, these data emphasize the importance of structured growth surveillance and tailored nutritional strategies, particularly during early infancy when growth velocity is highest and opportunities for intervention are greatest.

### Limitations

Several limitations should be acknowledged. First, the retrospective, single-center nature of the study limits the generalizability of the findings to other settings and populations. Second, although the overall sample size was acceptable, the relatively small number of infants in the normal birth weight group reduced statistical power for subgroup comparisons.

Third, the analysis focused on weight and length; head circumference was not available at most follow-up visits, and neurodevelopmental, cognitive, and metabolic outcomes critical long-term consequences of prematurity were not assessed. Fourth, feeding practices were recorded descriptively, but the lack of detailed quantitative nutritional data limited evaluation of their true impact on growth trajectories.

Finally, sex-specific growth patterns were not examined in detail, and potential confounders such as neonatal morbidity, illness severity, and socioeconomic factors were not fully adjusted for, all of which may influence postnatal growth. The mixed-effects models adjusted for sex and gestational age, but these additional confounders were not consistently recorded and could not be incorporated; residual confounding and selection or follow-up bias cannot be excluded. Because measurements were obtained at discrete windows, the ability to distinguish transient from sustained catch-up is limited. We also note that a single WHO reference was applied across the full period; Fenton or INTERGROWTH-21st standards may be preferable for the early neonatal period.

Despite these limitations, the study provides meaningful longitudinal insight into growth patterns and the timing of catch-up growth among preterm infants in a tertiary care context, contributing valuable data to an area where evidence remains limited.

## Conclusions

In summary, preterm infants exhibited significant early growth deficits compared with term infants, particularly among those born with very low birth weight. While weight catch-up occurred relatively early with substantial recovery achieved within the first year of corrected age, linear growth followed a slower trajectory, extending closer to 24 months. An observed pattern of more rapid weight gain was noted between 4 and 8 months of corrected age; given the observational design, this is described as an observed pattern rather than a proven developmental milestone or optimal intervention window. Although feeding practices varied, no clear association with catch-up growth was observed, and this should be interpreted as exploratory. These findings underscore the dynamic nature of postnatal growth in preterm infants and highlight the need for vigilant growth monitoring and individualized nutritional strategies during early infancy to support optimal long-term growth outcomes.

## Supplementary Information

Additional file 1.

## Data Availability

The data that support the findings of this study are derived from hospital/institutional medical records and are not publicly available due to patient privacy and confidentiality restrictions. The datasets are available from the corresponding author upon reasonable request and subject to institutional approval.

## Abbreviations

ANOVA: Analysis of variance
AR(1): First-order autoregressive
CA: Corrected age
CI: Confidence interval
FGR: Fetal growth restriction
GA: Gestational age
HC: Head circumference
IRB: Institutional Review Board
KSUMC: King Saud University Medical City
LBW: Low birth weight
NBW: Normal birth weight
REML: Restricted maximum likelihood
SD: Standard deviation
SGA: Small for gestational age
SPSS: Statistical Package for the Social Sciences
STROBE: Strengthening the Reporting of Observational Studies in Epidemiology
VLBW: Very low birth weight
WHO: World Health Organization
WLZ: Weight-for-length Z-score
