# Supplementary material for: Time to catch-up: postnatal growth trajectories in preterm infants at a tertiary care hospital in Riyadh, Saudi Arabia
Source: BMC Pediatr. 2026 Jun 24;26:844. doi: 10.1186/s12887-026-07223-5 (PMC13555970; doi:10.1186/s12887-026-07223-5)
Supplement: Supplementary file 1 [file 12887_2026_7223_MOESM1_ESM.docx]

**Additional file 1: STROBE Statement Checklist**

*Checklist of items that should be included in reports of cohort studies (von Elm et al., STROBE statement, 2007).*

| Section/Topic | Item No. | Recommendation | Reported on page/section |
| --- | --- | --- | --- |
| Title and abstract | 1(a) | Indicate the study's design with a commonly used term in the title or the abstract | Title page; Abstract (Methods) |
|  | 1(b) | Provide in the abstract an informative and balanced summary of what was done and what was found | Abstract |
| Introduction |  |  |  |
| Background/rationale | 2 | Explain the scientific background and rationale for the investigation being reported | Background |
| Objectives | 3 | State specific objectives, including any prespecified hypotheses | Background (final paragraph) |
| Methods |  |  |  |
| Study design | 4 | Present key elements of study design early in the paper | Methods: Study Design and Setting |
| Setting | 5 | Describe the setting, locations, and relevant dates, including periods of recruitment, exposure, follow-up, and data collection | Methods: Study Design and Setting |
| Participants | 6(a) | Give the eligibility criteria, and the sources and methods of selection of participants. Describe methods of follow-up | Methods: Study Population and Eligibility Criteria |
|  | 6(b) | For matched studies, give matching criteria and number of exposed and unexposed | Not applicable (not a matched design) |
| Variables | 7 | Clearly define all outcomes, exposures, predictors, potential confounders, and effect modifiers. Give diagnostic criteria, if applicable | Methods: Definitions; Outcome Measures |
| Data sources/measurement | 8* | For each variable of interest, give sources of data and details of methods of assessment (measurement). Describe comparability of assessment methods if there is more than one group | Methods: Data Collection; Outcome Measures |
| Bias | 9 | Describe any efforts to address potential sources of bias | Methods: Data Collection; Limitations |
| Study size | 10 | Explain how the study size was arrived at | Methods: Sample Size |
| Quantitative variables | 11 | Explain how quantitative variables were handled in the analyses. If applicable, describe which groupings were chosen and why | Methods: Definitions; Statistical Analysis |
| Statistical methods | 12(a) | Describe all statistical methods, including those used to control for confounding | Methods: Statistical Analysis |
|  | 12(b) | Describe any methods used to examine subgroups and interactions | Methods: Statistical Analysis (stratification by birth-weight category) |
|  | 12(c) | Explain how missing data were addressed | Methods: Statistical Analysis |
|  | 12(d) | If applicable, explain how loss to follow-up was addressed | Methods: Study Population (exclusion for <3 visits); Limitations |
|  | 12(e) | Describe any sensitivity analyses | Not performed |
| Results |  |  |  |
| Participants | 13(a) | Report numbers of individuals at each stage of study — e.g., numbers potentially eligible, examined for eligibility, confirmed eligible, included, completing follow-up, and analysed | Results: Baseline Characteristics |
|  | 13(b) | Give reasons for non-participation at each stage | Methods: Study Population; Limitations |
|  | 13(c) | Consider use of a flow diagram | Not included |
| Descriptive data | 14(a) | Give characteristics of study participants (e.g., demographic, clinical, social) and information on exposures and potential confounders | Results: Baseline Characteristics; Table 1 |
|  | 14(b) | Indicate number of participants with missing data for each variable of interest | Tables 2–6 (n reported per interval) |
|  | 14(c) | Summarise follow-up time (e.g., average and total amount) | Methods: Data Collection (follow-up to 24 months) |
| Outcome data | 15* | Report numbers of outcome events or summary measures over time | Results: Tables 2–6; Figures 1–2 |
| Main results | 16(a) | Give unadjusted estimates and, if applicable, confounder-adjusted estimates and their precision (e.g., 95% confidence interval). Make clear which confounders were adjusted for and why they were included | Results: Tables 1–6 (unadjusted; no multivariable adjustment) |
|  | 16(b) | Report category boundaries when continuous variables were categorized | Methods: Definitions (VLBW/LBW/NBW thresholds) |
|  | 16(c) | If relevant, translate estimates of relative risk into absolute risk for a meaningful time period | Not applicable |
| Other analyses | 17 | Report other analyses done — e.g., analyses of subgroups and interactions, and sensitivity analyses | Results: stratified analyses by birth-weight category (Tables 4–6) |
| Discussion |  |  |  |
| Key results | 18 | Summarise key results with reference to study objectives | Discussion (opening paragraphs) |
| Limitations | 19 | Discuss limitations of the study, taking into account sources of potential bias or imprecision. Discuss both direction and magnitude of any potential bias | Limitations |
| Interpretation | 20 | Give a cautious overall interpretation of results considering objectives, limitations, multiplicity of analyses, results from similar studies, and other relevant evidence | Discussion; Conclusions |
| Generalisability | 21 | Discuss the generalisability (external validity) of the study results | Limitations; Discussion |
| Other information |  |  |  |
| Funding | 22 | Give the source of funding and the role of the funders for the present study and, if applicable, for the original study on which the present article is based | Declarations Funding |
